# Scarcity-driven monetization of digital content

**DOI:** 10.3389/frma.2022.995202

**Published:** 2022-08-22

**Authors:** Vamsi K. Kanuri, Adithya Pattabhiramaiah

**Affiliations:** ^1^University of Notre Dame, South Bend, IN, United States; ^2^Georgia Institute of Technology, Atlanta, GA, United States

**Keywords:** content scarcity, paywall, media platform, newspaper, engagement

## Abstract

In the last two decades, the U.S. news industry has undergone significant disruption, which resulted in nearly a 66% drop in overall revenues. Such a monumental decline in subscription and advertising revenues has led news publishers to experiment with new revenue generation strategies. Some of these strategies, such as instituting a paywall on the newspaper's website and deploying a freemium business model have gained in popularity due to their promise of generating additional subscription and advertising revenue. However, these strategies limit readers' access to news, thereby contributing to news becoming a scarcer commodity. In contrast, alternative strategies such as reader-focused fundraising events aim to increase revenue organically by educating readers about the cost and value of quality journalism, with little implication for news scarcity. In this chapter, we survey several of these contemporary digital news monetization strategies with the goal of assessing the sustainability of scarcity-driven strategies. We offer conjectures about the conditions under which scarcity-driven strategies may be profitable relative to alternative monetization strategies and share some predictions about upcoming trends in the news industry.

## Introduction

In the last two decades, the U.S. news industry has undergone significant disruption. Once only accessible through traditional distribution channels such as print, radio, and television, news is now increasingly available through contemporary digital channels. The capabilities of these contemporary channels have enabled news organizations to adopt novel monetization strategies. Many of these strategies rely on limiting readers' access to news, which in turn has contributed to news becoming a scarce commodity. The goal of this article is to discuss such scarcity-driven news monetization strategies. Specifically, first, we contextualize the rise of scarcity-driven news monetization strategies. Second, we survey the most popular scarcity-driven news monetization strategies. Third, we discuss the social impact of scarcity-driven monetization strategies, beginning with a characterization of the specific demographic groups that are most likely to be influenced by such strategies. Last, we offer some predictions on the evolution of scarcity-driven monetization strategies.

To better understand the antecedents of scarcity-driven monetization of newspapers, we begin with a review of some key historical trends in the market for news. Out of all media businesses, the news publishing industry in the United States, in particular, has experienced significant disruption over the last two decades. Up until the dawn of the 21st century, news was predominantly distributed *via* print, radio, and television channels. Consumers who were willing to pay a price for accessing news on a certain medium typically received full access to news on that medium. Traditional subscription-based monetization policies focused on granting consumers access to news using either a time dimension (e.g., 6-month print subscription contracts) and/or a product dimension (e.g., subscription to newspapers on weekdays/weekends). Subscription contract averse readers also had the option of purchasing individual copies of the newspaper. A distinctive characteristic of the pre-digital news monetization strategies is that paid readers were offered unrestricted access to all the news stories featured in the newspaper.

With the commercialization of the internet, starting in the early 2000's, newspapers started slowly but surely recognizing a shift in consumers' attention toward digital media for most of their information needs, including news. The upside of serving a digitally inclined audience seemed potentially promising, especially from a customer retention and loyalty standpoint. As newspapers started moving sections of the newspaper online, the ease of tracking consumer engagement with different types of news content presented them with additional and highly lucrative opportunities for customer segmentation. While alternative forms of enabling/restricting access to specific news content (e.g., restricting access to sections of the newspaper–say sports or business news, only for paid subscribers) were rare prior to the arrival of the internet, it quickly started gaining in prominence thereafter. The emergence of digital channels as the dominant conduit for public opinion expression saw newspapers rushing to incorporate features such as reader board communities, discussion groups, and opinion editorials on their websites. The allure of accessing content for “free” coupled with an option of connecting with like-minded community members saw a steady shift in consumer attention in favor of digital news.

Nonetheless, motivated partially by their reliance on the success of the print newspapers (where advertising rates were still several 100 times higher than the digital counterparts)^1^, news organizations doggedly maintained a firm belief that the trend in readers' migration toward digital was only temporary. The news industry experienced all of the teething troubles that are common to industries looking to break into new markets/channels of operation. Moreover, during the early days of digital newspapers, with internet speeds still slow, websites were often slow to load and difficult to navigate, leading to poor user experience. News publishers took this opportunity to promote the unique virtues of the print newspaper (e.g., its consistently high production standards, and touch and feel experiential aspects) relative to digital news, to stay competitive.

Further, as local monopolies operating in well-defined circulation territories, local newspapers, in particular, were laser-focused on compiling projections of the size of their installed base of readers with a view of appealing to print advertisers. With a sizable installed base of mostly loyal (i.e., paying) print subscribers, newspapers were loath to question the relative importance of the print channel for their survival. While the costs associated with producing quality journalism were not trivial, news organizations heavily relied on the market's appetite for such content as a basis for their sustainable operations and profits.

At the same time, starting around 2005, several concomitant developments introduced pivotal shifts in newspapers' marketplace for reader and advertiser attention: the rising prominence of social media and craigslist. These avenues, which seemed novel at the time, prompted a substitution of consumer attention away from newspapers, posing direct consequences for the perceived attractiveness of display advertising and classifieds hosted in newspapers. Advertisers, in fact, were particularly quick to spot such shifts in consumer attention, leading to a reallocation of advertising budgets away from newspapers and toward these alternative avenues. This led to a steep drop in print newspapers' advertising revenues and rates. In fact, according to the PEW research center, the total advertising revenue generated by U.S. newspapers has plummeted from $48.67 billion in 2000 to an estimated $11.09 billion in 2020–a staggering ~77% decline (Barthel Worden, 2020). During the same time, print circulation declined from 55.77 million in 2000 to an estimated 24.30 million in 2020 for the weekday product, and 59.42 million in 2000 to an estimated 25.79 million in 2020 for the Sunday product (Barthel Worden, 2020).

By 2010, most U.S. newspapers had come to terms with the reality that readers' migration to digital channels is perhaps more permanent than imagined. The precipitous decline in print advertising and subscription revenues has since forced many U.S. newspapers to cut back on newsroom expenditures by laying off editorial staff and journalists. In addition, some newspapers such as The Times-Picayune in New Orleans, Oneida Daily Dispatch in New York, and Washington Times-Herald in Washington even scaled back their print production by reducing the number of weekdays they delivered the print newspaper. Although these cost-cutting strategies were successful in freeing up some resources and helping these newspapers stay afloat, publishers were fully aware that those strategies were only a temporary measure applied to resolving a long-term problem (Edmonds, 2015). Some newspapers additionally experimented with a switch to digital-only operations to save on production and distribution costs. It is against this backdrop that news publishers began exploring alternative monetization strategies.

## The rise of scarcity-driven monetization strategies

The inevitable shift from free to fee has prompted content platforms to adopt creative ways to monetize their online content. Readers are sensitive to such content monetization strategies. For instance, prior research has argued that instituting a paywall negatively influences the digital engagement of both light and heavy readers (Pattabhiramaiah et al., 2019). At the same time, paywalls can potentially drive readers to the print product, and therefore have a positive influence on print circulation (an aspect that has been termed the “spillover effect” of digital monetization). For several decades leading up to 2010, print newspapers made up the lion's share of publisher revenues (between 60 and 80%). However, in recent times, the balance has started to shift in favor of digital news. Recognizing the importance of preserving the print subscriber base, to the extent possible, most newspapers in the United States now bundle free access to digital newspapers with print subscriptions. While it is well-known that consumers rarely take full advantage of products especially when they are offered for free (Shampanier et al., 2007), bundling free access to digital news with print subscriptions has been shown to provide a tangible subscriber retention benefit for publishers (Pattabhiramaiah et al., 2022). In fact, print subscribers, who avail of such unlimited (free) access to digital news, end up delaying their subscription termination decisions, thereby contributing 7–12% higher revenues for newspapers.

Readers' preference for news is also shaped by the subscription bundles offered by the content platforms. Content bundles allow firms to cater to the heterogeneity in readers' preference for the channel used to consume news. Paid content that was once distributed exclusively *via* conventional media avenues such as print, radio, and television is now also available through contemporary digital formats such as websites, smartphone apps, and tablet apps. Moreover, these contemporary formats are much more versatile, making it much easier for news to be deployed using different versioning strategies to consumers. For example, digital paywalls have made it a straightforward proposition for newspapers to deliver digital news content through a “restricted-access” version (e.g., up to 20 free articles per month before charges) and an unrestricted version (full access to subscribers). There is increasing evidence that readers are willing to pay for the bundles that offer them the most flexibility with consuming content. For example, readers' willingness to pay has been shown to be significantly higher for multi-format bundles (i.e., bundles comprising some combination of print, online, smartphone, and tablet access) when compared to pure component bundles (Kanuri et al., 2017). Additionally, the likelihood of a reader becoming a paid subscriber has been shown to significantly increase when the menu features a pure or mixed bundle as opposed to just pure components (e.g., a menu comprising online-only, print-only, or smartphone-only access). These patterns underscore the fundamental downstream consequences of bundled pricing and their role in influencing the scarcity in consumers' news access: when consumers are forced to choose between a pure components bundle or forgo consumption, the downstream consequences of a sizable base on consumers opting into the latter category is stark. In this way, bundling may create adverse externalities for readers' access to news.

As the public's taste for bundled offerings drops, the market inevitably starts reexamining the optimality of bundling. As has been argued in prior research, readers' content consumption preferences are influenced by the availability of unbundled news content of different types. Unbundled content allows platforms to cater to differences in readers' taste for content in different sections of the newspaper: comics, local news, business news, etc. Readers differ in their willingness to pay higher prices for some types of news content than for others (Graybeal and Hayes, 2011). At the same time, readers may also be sensitive to visible drops in news quality triggered by the unbundling of news content strategy. For instance, using a game-theoretic model, Bisceglia (unpublished)^2^ demonstrates that an increase in reader preferences for a specific type of news (e.g., sports news) might motivate publishers to redirect their resources in favor of those popular sections and deliver richer content specifically in those sections. Nonetheless, recognizing that content development resources are finite, a perceived drop in the quality of news content in other sections of the newspaper could easily motivate readers to switch to competing content providers. These patterns may further contribute to a scarcity in consumers' access to news provided by newspapers.

Last, governmental policies can also (sometimes unintentionally) contribute to the scarcity of online content by restricting its access. For instance, Article 15 of the EU Digital Single Market directive required Google to drop links to European news sites because the directive found Google's use of news headlines to identify the linked story as an act of copyright infringement (Lemley, 2021). This policy has resulted in a sharp decline of as much as 50% in user visits to European newspaper websites because users were unaware of the stories reported by these newspapers.

## Non-scarcity-driven monetization strategies

A continued shift in reader preferences and the accelerated rates of decline in subscription and advertising revenues have prompted media firms to think beyond the news product and identify newer ways to upsell to existing readers and acquire new readers.

One such alternative revenue source that has quickly emerged as a popular monetization strategy is niche events. Niche events are themed programs that are tailored to the preferences of a segment in the media firm's target market. Some examples of niche events include Vogue's Wedding Show, GQ magazine's Comedy Extravaganza, Conde Nast's Russia Digital Day, Atlantic Media's Aspen Ideas Festival, The New York's Vulture Festival, and New York Culinary Experience, and The New York Times' Conversation.

Niche events are attractive to media firms mainly because of their appeal to advertisers. Events open a whole new range of possibilities for advertisers for showcasing their products and services, which in some cases, offer better ROI than print and digital advertising within the news product. For instance, media firms are now selling advertising in pre-event promotions, pre-roll advertising on videos of the event, sponsorship of streaming video from the event, event signage, booths at the event, and 60-s pitches to the niche audience at events (Lutz, 2021).

Another reason why events have garnered popularity is their ability to monetize readers' exclusive access to popular personalities, and their ability to personalize entertainment and information to readers. For example, the infamous events referred to as the Salons, organized by the Washington Post offered lobbyists exclusive access to political personalities and the newspaper's executives for anywhere between 25,000 and $250,000 (Shafer, 2009). The Aspen Ideas Festival costs as much as $3,000 for a four-day pass^3^ Other niche events such as the New York Ideas conference drew 815 people at $149 each and Start-Up City: Miami enticed 700 people at $75 a ticket (Lutz, 2021).

Another attractive feature of events is their ability to generate indirect network effects for media firms. Events create a sense of community membership, potentially incentivizing readers to subscribe to the news service offered by the media firm (Scruggs, 2020). For example, during the COVID-19 pandemic, virtual events were the only type of gathering most readers were able to attend. Through the small-group discussions facilitated by features such as the virtual meeting rooms in Zoom, virtual events were able to cultivate a sense of belongingness among the readers, which subsequently incentivized readers to become paid subscribers at the newspaper (Scruggs, 2020). As such, many media companies including The Next Web, Bloomberg Media, and the Financial Times have reported a marked increase in paying subscribers following live events during the pandemic (McCarthy, 2020). Media companies hope that such an increase in subscribers could in turn drive up advertising revenues in the long term.

## Realizing the impact of scarcity-driven monetization strategies

Academic research has long argued that firms can strategically employ a tool for boosting consumer willingness to pay: product scarcity (Cialdini and James, 2009). But how precisely can newspapers influence consumers' willingness to pay? To answer this all-important question, the first step involves understanding the different attributes that shape readers' utility function as it relates to news consumption, which in turn influences their willingness to pay for news.

### Reader demographics

In our daily lives, demographics play a significant role in shaping the public's preferences for products, including their news consumption preferences. Journalism research has argued that age, income, education, and gender all directly influence consumers' willingness to pay for news (e.g., Chyi, 2005, 2012; Goyanes, 2014). In fact, younger consumers, who are typically more adept at using new media channels such as online and mobile apps, are believed to be more willing to pay for news in these formats (Chyi, 2005, 2012). Younger consumers are also more accustomed to paying for online products in general, increasing their affinity and willingness to pay for online news. In contrast, older individuals, who are used to the content formatting and tactile experience of print newspapers have a higher willingness to pay for offline (or print) news. George (2008) studied the effect of the internet (as a proxy for increased consumer propensity to access news content online) on print newspapers in the US market and found that increased internet penetration levels predominantly influence young, educated urban readers away from daily print newspapers. She also provides evidence for content reformulation strategies that newspapers likely adopt in response to the internet, with a greater emphasis on minorities, education, crime, and investigative reporting in order to differentiate from online newspaper content.

Unlike age, income has garnered mixed findings in the literature. On the one hand, researchers have hypothesized and found that income is a significant driver of willingness to pay for online content because of its logical relationship with an individual's ability to pay. In addition, higher-income consumers value their time more because of its opportunity cost (Stigler, 1961). Hence, to mitigate the opportunity cost of searching for online news, higher-income consumers are more likely to pay for news than lower-income consumers. Yet, other studies have found a negative relationship between income and willingness to pay for online news (e.g., Chyi and Yang, 2009; Goyanes, 2014). One plausible reason for this counterintuitive finding is that higher-income individuals view online news as inferior goods (Punj, 2013). The perception that online news is less differentiated could lead higher-income individuals to view online news as an inferior good, in turn reducing their propensity to pay for online news. Regardless, an individual's income also appears to significantly influence their news consumption preferences.

Furthermore, a newspaper reader's education level also tends to drive their news consumption preferences. Individuals that have attained higher levels of education have a greater need for “smart” content because those individuals are more likely to have the expertise and fluency to derive greater benefit/information value from such content (Punj, 2013). Moreover, the level of education is directly correlated with the need for new knowledge and information. Therefore, the level of education could also directly influence an individual's propensity to consume and pay for news.

Lastly, prior research has reported mixed findings with respect to the influence of gender on willingness to pay for news. For instance, because women are more likely to emphasize the social aspect of information (Slyke et al., 2002), some researchers have predicted women to have a higher willingness to pay (e.g., Punj, 2013, 2015). However, other studies have found no relationship between gender and an individual's willingness to pay (Chyi, 2005, 2012; Goyanes, 2014).

Notably, the focus of all these studies is on examining the role of demographics in influencing consumers' willingness to pay for news. In light of these findings, while it is intuitive that demographic differences might reveal some asymmetries in the response of newspaper readers (of different age, gender, and education profiles) to scarcity-driven monetization strategies, providing formal evidence to support the existence of such patterns is still a promising topic for future inquiry.

### Access to news

Another key determinant of consumers' willingness to pay for news media is the availability of alternative news sources. Regardless of the credibility of a news publisher, individuals tend to have a lower preference for consuming news from that publisher if they are able to access the same news story free of charge from another outlet. In other words, media accessibility can affect the gratification that individuals derive from news consumption and therefore, affects their propensity to pay for news (Van der Wurff, 2011). The proliferation of free sources of news has created an illusion among readers that news is a commodity. Several surveys reveal that readers think it is unfair for service providers to charge money for online news because they derive revenue from advertisements (Chyi, 2005). Although online advertising is often less lucrative to publishers than customers perceive it to be, the customer mentality of expecting all services and information to be available to everyone at zero cost (“free mentality”) seems at least partially responsible for driving a lack of consumer willingness to pay for news (Chyi, 2005; Pauwels and Weiss, 2008; Kanuri et al., 2017).

Psychologists note that the free mentality can have ripple effects. As users' default expectation is for online content to be available at no cost, users who attach a high value only on content available for “free” tend to undervalue the tradeoff between the cost of news and its associated quality (Niemand et al., 2019). These irrational consumer expectations suggest a suboptimal pricing equilibrium for news publishers wherein news should only be offered for free to readers. Some studies have argued that newspapers can overcome such problems by offering exclusive content. Exclusivity can induce a feeling of scarcity that could potentially drive up the demand for news (Mensing, 2007). In fact, some practitioners attribute the success of news publishers such as The Wall Street Journal and the Financial Times to the quality and exclusivity of their content offering (Sjøvaag, 2016).

Individuals derive utility from being able to access the type of information they seek through the device of their choosing. For instance, it is natural to expect that consumers are less interested in paying for content aired at a time when they are unavailable to consume it (e.g., midnight). The same logic holds true for news content. In fact, content posted on a newspaper's dedicated social media page was shown to receive different levels of engagement based on the time of day when the stories were posted (Kanuri et al., 2018). For example, sports stories posted on social media (e.g., articles pertaining to a local NFL team) appeared to receive the lowest engagement in the morning because individuals are more likely to be interested in consuming local and national news stories in the mornings. Thus, scarcity-driven monetization strategies that make news available to readers when they are less interested in accessing it could also negatively affect their willingness to pay for news.

### Correlated behaviors

Individuals do not exhibit siloed preferences. Their day-to-day behaviors and possessions tend to have a spillover effect on their different tasks/activities. Accordingly, within the context of news consumption, the devices individuals own and their other content consumption preferences could dictate their propensity to consume news. For example, multi-device ownership has been shown to be related to an increased likelihood of paying for news among consumers (Chyi and Chadha, 2012). A recent PEW study indicates that more people in the United States now own mobile devices such as smartphones, e-book readers, and tablets, compared to before^4^. With so many gadgets permeating the market and offering a myriad of platforms and options for people to access media, news businesses have begun targeting this fragmented market by offering news in multiple formats. This cross-media strategy (also referred to in the industry as a “360-degree strategy”) has allowed individuals who own multiple devices to subscribe to news bundles that allow them access through these different devices.

Similarly, an individual's general media consumption behaviors and certain purchase behaviors are known to be positively related to their news consumption tendencies. Consumers who consume news on television are more likely to also consume news from print and online media (Leung and Wei, 1999; Dutta-Bergman, 2004). Similarly, an individual's news consumption behavior is positively associated with their eBook readership, Twitter usage, videos/TV content, software ownership, and app purchases (Goyanes, 2014). Selective exposure theory can explain these behaviors. This theory posits that individuals orient themselves to specific stimuli in their environment because of their enduring interest in those subject areas (Petty and Cacioppo, 1986). For instance, an individual who is interested in sports news might read the sports section of the newspaper in the morning, then tune into a sports radio channel on his way to work and read an opinion piece on a recent game on a newspaper's website thereafter. Therefore, positive correlations in preferences for information access *via* different media could also drive an individual's overall news consumption preferences.

On the other hand, some studies have questioned the role of such positive correlations in media preferences in driving news consumption preferences. This contrarian viewpoint argues that individuals have a fixed amount of discretionary time that can be allocated across different media, on any given day. Any excess time allocated to one media platform (say e.g., social media) is expected to come at the expense of content consumption on other channels. For example, Jang and Park (2016) observe substitution patterns among paper, television, and computer use. Regardless of whether content consumption preferences across media serve as a complementary or substitutive to news consumption on newspapers, consumers' propensity to pay for news appears to be directly affected by their preferences for content available on other non-newspaper media. Therefore, it is critical for newspapers to account for cross-platform synergies while designing scarcity-driven monetization strategies.

### Content differentiation

One key aspect of news that can be expected to affect an individual's propensity to pay for access to news on news websites is the differentiation of news content available from other sources. Readers arguably value differentiated content due to its ability to offer original ideas and/or exclusive information. A clearly articulated differentiation statement allows the firms to create brand loyalty among their customers (Chaudhuri and Holbrook, 2001; Sreenivasan et al., 2016). Therefore, a unique differentiation proposition effectively insulates publishers from their competitors. For instance, uniqueness may build a sizable entry barrier for other competitors to overcome (Porter, 1980) and offers publishers a sustained source of competitive advantage over other publishers–e.g., in the form of higher market shares (Stahl and Maass, 2004).

Content differentiation could also help newspapers battle the perception that news is a commodity (Picard, 2009). The proliferation of the internet has indeed exacerbated perceptions of news being a commodity among the readers–especially because accessing news in digital environments usually entails very low switching costs. In fact, product performance and success are adversely impacted when consumers actively switch between competitor websites (a practice referred to as “multihoming” in the academic literature; Cennamo et al., 2018). On the other hand, effective brand differentiation can materially boost consumer willingness to pay (Srinivasan et al., 2005). Hence, content differentiation could help newspapers better justify scarcity-based monetization strategies to their readers.

### Content personalization

The ability of content creators to cater to individual needs and tastes can also influence readers' preferences for paid content (Lin et al., 2014). Content creators recognize this need and frequently deploy recommendation systems on their websites. Recommendation systems adapt the content, delivery, and arrangement of content on the website to individual users' explicitly registered and/or implicitly stated preferences (Thurman and Schifferes, 2012). These systems directly address the choice overload problem that readers face on news websites. News websites typically contain a large amount of information. However, the majority of readers have finite, and often quite narrow content needs. With the increasing availability of rich clickstream data on readers' consumption of news on websites, recommendation systems on news sites are getting rapidly adept at recognizing these narrow needs–at the customer segment level, or even at an individual reader level. Computational advances such as collaborative filtering and content-based filtering have enabled news recommendation algorithms to exploit both the similarities and uniqueness in the behaviors of users of different activity profiles to offer personalized content recommendations in real-time. While such content targeting algorithms differ in their approach (collaborative filtering looks at the similarity between users and between items, and content-based filtering uses text analysis for matching users with the types of stories they have engaged with in the past), they promise a sustained source of engagement benefit for news publishers.

Personalized recommendations can significantly reduce readers' search costs by helping readers identify articles they are most likely to enjoy reading, thereby boosting the reader's valuation of the content provider. Lots of studies have noted the synergistic relationship between personalization and usage. The rationale is that personalization promotes autonomy (i.e., readers' voluntary inclinations for engaging in an activity), increases persistence, and fosters an emotional bond that individuals seek in everything they do (Oulasvirta and Blom, 2008; Thurman and Schifferes, 2012). Relatedly, Google reported that content personalization based on collaborative-filtering algorithms resulted in a 38% increase in clicks on stories (Das et al., 2007, p. 279). Thus, personalization aids can help readers better appreciate a news provider's source of content differentiation.

### Price sensitivity and sensitivity to other supply-side instruments

Price plays an especially crucial role in driving consumers' content consumption preferences. For many years, content creators primarily charged readers only for content published in their print product and gave away their online content for free. As noted before, shifts in the media landscape saw readers shift their attention away from newspapers toward modern media sources, prompting the inevitable outbound migration of advertisers. The resulting decline in print subscription rates and advertising revenues has prompted firms to carefully reprioritize and role of online as a chief source of revenues. Nonetheless, charging for online content is difficult owing to a long-held belief among consumers that digital content should be free (Lambrecht and Misra, 2017). The decline in readers' preferences for reading newspapers has been accompanied by an increased sensitivity to price changes. Additionally, charging for online news is challenging because general-interest news stories in an online setting are seen as having close substitutes, often also available for free (Picard, 2016). These patterns promote the widely held view that charging for online news will result in a significant decline in readership (Chyi, 2005; Chiou and Tucker, 2013).

Readers are also known to be sensitive to both acquisition and retention-focused price promotions offered by content creators. As two-sided platforms, newspaper firms rely on two interlinked sources of revenue–reader subscriptions and advertising. In the heyday of newspapers, it was optimal to subsidize readers' access to news with a view of attracting higher advertising revenues (that were expected to offset the lower subscription revenues resulting from price promotions). However, offering price promotions to readers can have unintended consequences in the long run for content platforms (Kanuri and Andrews, 2019). Specifically, when content platforms offer price promotions, they run the risk of lowering their readers' internal reference prices. Reference prices denote the price that users see as “fair” for the given product/service (Kalyanaram and Winer, 1995; Xia et al., 2004). Prices higher than the reference price risk putting readers in a frame of mind wherein they start questioning why they should pay a price higher than what they see as “equitable” (Kahneman and Tversky, 2013). Such situations can negatively influence purchase decisions by discouraging readers from renewing their subscriptions. Readers may become sensitive not only to prices but to the availability of price promotions. For example, frequent price promotions can lower consumers' reference prices (Alba et al., 1999) and inadvertently train them to avoid purchasing the item when it is not on promotion. Taken together, it is natural to expect that readers' reactions to firms' scarcity-based monetization strategies is a function of their price (and price promotion) expectations.

### Political slant

Readers exhibit a strong preference for consuming news that conforms with their ideologies and beliefs (Gentzkow and Shapiro, 2010). This preference appears to be driven by their fundamental need for avoiding cognitive dissonance or negative feelings prompted by being confronted with information that questions any pre-existing beliefs (Festinger, 1957). Accordingly, readers tend to naturally gravitate toward news sources that align with their ideologies (Garrett, 2009; Kitchens et al., 2020). The selective exposure theory also purports that people are more likely to consume opinion-reinforcing news as opposed to opinion-challenging news (Frey, 1986). Consistent with this view, readers tend to favor content platforms that adopt algorithmic filtering of content that ensures a greater likelihood of encountering content that aligns with a priori beliefs presumably because the rest is filtered out algorithmically (Van Alstyne and Brynjolfsson, 2005; Levy, 2021).

However, personalization based on readers' political preferences can have various adverse consequences for both the readers and society. For example, readers of political blogs can become more ideologically segregated and more ideologically extreme than non-readers (Lawrence et al., 2010). Such polarization in reader preferences may lead to social fragmentation and intellectual isolation, both of which are counterproductive to society (Sunstein, 2002; Pariser, 2011). Furthermore, intellectual isolation can also create epistemic bubbles wherein personal viewpoints remain unchallenged and untested (Pariser, 2011). Ideological segregation can also result in confirmation bias, wherein people continue visiting only those media outlets that host information congruent with a pre-existing belief or notion; each incremental encounter with such information only solidifies the reader's desire to discount divergent viewpoints, resulting in an echo chamber. Such behaviors can result in extreme polarization of society and help propagate the spread of misinformation and fake news (Chaffee and Metzger, 2001; Bennett and Iyengar, 2008; Del Vicario et al., 2016; Allcott and Gentzkow, 2017).

Recognizing the dire nature of these consequences, what if anything, can news publishers do in the way of helping alleviate their incidence? On the one hand, it seems promising that ideological polarization can be mitigated through random variation in the exposure to ideological content: randomly exposing individuals to counter-attitudinal news decreased consumers' negative attitudes toward an opposing political party (Levy, 2021). On the other hand, such mitigation measures may seem too optimistic (Kitchens et al., 2020). Specifically, one downside of exposing people to divergent viewpoints may be that it can catalyze a polarizing backlash that further hardens any pre-existing ideological positions (Bail et al., 2018). Furthermore, while readers spend more time on media outlets that host information that conforms with their political leanings, they appear to also engage in considerable cross-partisan media exposure anyway (Cardenal et al., 2019). This suggests that the public's mere exposure to ideologically dissimilar content may not effectively mitigate polarization. Regardless, there is unequivocal evidence suggesting that readers derive tangible psychological benefits from the political slant adopted by content platforms (Chiang and Knight, 2011), implying that the degree of political slant may have implications for the success of scarcity-based monetization strategies employed by newspapers.

### Channel of news delivery

Usage situations also play a critical role in readers' news consumption preferences because they dictate readers' perceptions of products as substitutes or complements. The substitution-in-use (SIU) theory postulates that intended usage determines whether individuals treat products as substitutes or complements. It assumes that products are a means of achieving usage-related goals. When two products are appropriate for the same usage situation, they are perceived as providing similar benefits and are therefore considered substitutable (Ratneshwar and Shocker, 1991). When the products have distinctive uses, however, they are viewed as more dissimilar and are less likely to be viewed as substitutes.

It is plausible for consumers to view online and print channels as substitutes or complements. However, within the news context, most studies support the narrative that readers view their experience with print and digital newspapers as complementary. For instance, Chyi (2005) and Goyanes (2014) note that subscription to print news increases consumers' willingness to pay for online news because the online channels allow the print readers to access news through the online channel when the print paper is not available (e.g., while the readers are traveling or the reader is in another location such as an office). Similarly, Gentzkow (2007) argues that newspaper readers view print and online news channels as complements mostly because online news offers substantial incremental welfare benefits to readers, such as the access to news in real time/more updated news. Taken together, the relationship between print and digital news as visualized by readers can impact scarcity-based monetization for newspapers.

### The role of advertising

Accounting for network effects is critical to evaluating the success and failure of firms that derive revenues from multiple sources (Rysman, 2009). Network effects, or indirect network externalities, refer to a setting wherein the participation of at least one type of agent (e.g., newspaper advertisers) depends on other types of agents (e.g., newspaper readers). If a newspaper is popular among readers, this generates a type of positive feedback that increases advertisers' desire to advertise with that newspaper because they can reach a more sizable base of newspaper readers (Gabszewicz et al., 2005; Rochet and Tirole, 2006). The agent who values the presence of the other type of agent usually is charged the premium price. The premise behind this monetization applies in many other settings: men pay a stiffer cover charge than women to access night clubs, merchants pay higher fees to the American Express platform than consumers etc. In the case of newspapers, advertisers traditionally paid premium advertising rates, which helped keep subscription prices low enough to attract more readers. Similarly, Kaiser and Wright (2006) find evidence for asymmetric rent extraction from advertisers (in the German magazine industry) which provides consumers a subsidy in the cover price of the magazine–this is mainly because advertisers value access to consumers higher than consumers value advertising in magazines. However, the feedback/network effect between the different types of agents need not always be reinforcing/positive. Furthermore, its strength may also change over time. In settings where readers are increasingly averse to advertising (e.g., on live television), the price elasticity of advertising elasticity can increase (Wilbur, 2008).

Shifts in the advertising landscape over the last decade have taken a severe toll on newspaper revenues. While advertising contributed 87% of the revenue contribution per reader in 2006, its share dropped to 69% in 2011, to under 50% for the first time in 2020 (PEW Research center analysis)^5^. The increasing attractiveness of competing advertising options such as Craigslist, Google, and Facebook contributed the lion's share of print newspapers' revenue drop. Prior research has shown a direct association between craigslist's entry and average decreases of 5.7% in circulation shares and 3.5% in ad display rates due primarily to decreased prices (as high as 18.5%) for newspaper classified ads that imply increases of up to 3.6% in newspaper subscription prices (Seamans and Zhu, 2014). Building on this idea, other studies have shown that the stiff competition faced by newspapers for advertising dollars drove over 90% of the subscription price increases faced by consumers (Pattabhiramaiah et al., 2018).

Nonetheless, a part of the decline in print newspaper advertising over the last decade is also likely driven by the rising dominance of the newspaper's own online advertising format (Sridhar and Sriram, 2015). Academic research has actively sought to examine the relationship between online and offline advertising environments. Goldfarb and Tucker (2011a) use data on advertising bans in the alcohol advertising industry to study their effects on the effectiveness of online advertising in the regions affected by these bans. As the authors find a significant increase of 6% in online advertising effectiveness (operationalized as an increase in consumer self-report product purchase intent/product favorableness measures in states where out-of-home advertising of alcohol was banned) compared with 2% in states that did not have bans, they conclude that online advertising has higher effectiveness in these states. Focusing on supply-side reactions, other studies have documented increases in prices of (online) search ads when offline ads are banned (Goldfarb and Tucker, 2011b). This empirical setting is unique because it allows for a clean quantification of increases to advertising prices at search engines (cost per click paid) for search terms used by lawyers when they are unable to contact their clients by mail. Different studies have tried to further this inquiry. Building on Bergemann and Bonatti (2011)'s theoretical framework, Gentzkow (2014) documents that the price of consumer attention is higher online than it is offline. His calculations show that, in 2012, newspaper publishers earned approximately $1.57 per hour of reader attention in the print channel, substantially smaller than its digital counterpart of $4.24 per hour. This gap is likely to have only worsened since.

In light of these patterns, media publishers have been aggressively seeking new avenues for boosting online advertising revenues. Newspapers, specifically, are increasingly hosting sponsored content (SC), a type of native advertising that resembles editorial information but possesses predominantly commercial intent, on their websites. Such sponsored content is known to achieve higher click-through rates than say (cluttered) banner ads. The new form of advertising appears increasingly attractive to advertisers looking for “low touch” and less intrusive messaging options. Additionally, sponsored content is routinely co-created by advertisers' and the publisher's specialized journalistic groups, which affords publishers better control over the content. Nonetheless, hosting sponsored content is risky for publishers. First, because the headlines of sponsored stories closely resemble those of editorial news, consumers may be easily misled into clicking on the former expecting to consume the latter. Recognizing the importance of preventing such deception, the Federal Trade Commission mandates that publishers disclose the identity of native advertising with a noticeable label (e.g., “sponsored” or “ads”). Nonetheless, it is not clear that such labels suffice for helping preserve the consumers' readership experience. Chae and Pattabhiramaiah (2022) study the economics for publishers from hosting sponsored stories alongside editorial news. They show that readers exposed to sponsored content become less engaged with the news website over time. This appears to be driven by readers feeling deceived by the headlines of commercially oriented (sponsored) stories when they resemble those of editorial news articles. However, the harm appears to be somewhat short-lived: readers exposed to sponsored content do not seem more likely to drop their newspaper subscriptions. This implies that publishers can be less concerned about the longer-term opportunity costs of hosting sponsored content (i.e., subscription revenues.) In this way, appropriately priced sponsored content could help achieve net positive economics for news publishers looking to boost revenues.

It is increasingly critical to understand the role that advertising plays in the news production process. Beattie et al. (2021) document the role of advertising incentives in driving media bias in the publishing industry. The authors show that newspapers are less likely to cover (potentially damaging) information pertaining to safety recalls from brands that regularly advertise with them. It is no surprise that editors actively monitor click performance in deciding what types of news to provision on their websites (Sen and Yildirim, unpublished).^6^ With the increasing availability of bigger and better quality data, newspapers are increasingly resorting to algorithms for the news curation task. Claussen et al. (2021) note that algorithmic recommendations may also present some downsides in contexts where there are large gaps in the information available for news curation. They suggest that human editors aided by algorithms are invariably better able to preserve consumer engagement than algorithms, or news editors working alone. Taken together, this body of research paints a mostly optimistic picture for newspapers looking for creative solutions for improving both subscription and advertising revenues.

Over and beyond the newspaper's content provisioning problem, understanding how different types of readers (e.g., those arriving directly to the news website vs. referred by a search engine or social media website) may engage with news is also important. Traffic referred from external websites to newspapers is estimated to be roughly a little more than half as valuable as direct traffic, in revenue terms (Deloitte, 2019). The majority of news publishers in the United States currently adopt what has been termed a “soft paywall” strategy, allowing users arriving to the website from social media and google search to continue consuming news even after they may have encountered a paywall stop page. By restricting access to all externally sourced traffic, news publishers risk both lower advertising revenues (those who encounter paywalls and likely stop reading news contribute no further ad revenues) and news becoming an even scarcer commodity. This highlights the tradeoff between reach vs. (economic) reward linked to newspapers' intentions for posting news stories on social media, or permitting externally referred traffic to access paywalled content, thereby impacting the scarcity of news to society.

### Payment strategies

Finally, the choice of the payment mode that news publishers may make available to readers is likely to also impact their news consumption preferences. Growing privacy and data breach concerns have left readers somewhat hesitant to share credit card information online for accessing online services. Moreover, some reader segments (e.g., older readers and readers in developing countries) are yet to fully embrace the convenience that online payment systems offer (Zhang and Nguyen, 2004). Therefore, some reader segments (e.g., older consumers) may strongly prefer that content platforms continue offering traditional payment modes, no matter how archaic they might seem (e.g., mailing a check, issuing money orders, etc.). On the other hand, the rising adoption of cryptocurrency and rapid money transfer applications, such as CASH and VENMO apps, for online payments is forcing content platforms to consider adopting contemporary payment modes for content access. Due to the ease with which they adopt and embrace new technologies, younger consumers may be more sensitive to the availability of these payment options than older consumers may be. Nevertheless, the modes of payment can also potentially influence readers' utility for consuming online news. Therefore, newspapers' payment format choices also likely impact the success of their scarcity-driven monetization strategies.

## The future of scarcity-driven content monetization strategies

There is little doubt that newspapers in the United States are facing the greatest threat in their history. Their subscription and advertising numbers are growing grimmer with each new report. While the aging print reader base and migration of print readers to online news sources is part of the problem, the reallocation of print advertising budgets to modern digital avenues poses an arguably bigger threat to the survival of legacy (advertising-reliant) media firms.

While these trends augur poorly for the survival of newspapers, there appears to be a silver lining. The precipitous decline in print revenues has forced the majority of the U.S. newspapers to adopt a digital-first mindset in all of their ongoing initiatives and be more deliberate in transitioning print customers to their own digital channels, before they leave en masse to other competing free sources of information available online. Additionally, the proliferation of internet use and the accelerated adoption of digital technologies have increased consumers' familiarity with online monetization strategies. Moreover, the success of OTT (over-the-top) digital video streaming services supports the view that consumers are now realizing that unique and well-differentiated digital content costs money.

All of these trends bode well for the adoption of scarcity-based news monetization strategies. While these strategies rely on restricting readers' access to content (prior to their subscription), there is growing evidence that scarcity-based monetization strategies can result in tangible boosts in revenue. For example, The Information, one of the largest newsrooms in the technology sector, has attracted more than 20,000 subscribers who are willing to pay $399 a year for accessing its paywalled content. The New York Times (NYT), LA Times, and Washington Post (among other well-known news organizations) have all reported success with the paywalls (Pattabhiramaiah et al., 2019). Similarly, Substack, a popular content hosting platform, has succeeded in amassing over 25 million readers, of whom over a million are paid subscribers. It is impressive that the majority of content contributors who have managed to gain subscribers, including substack, have done so by appealing to their readers' appetite for differentiated content offering. All of these examples offer evidence that at least some reader segments have warmed up to the idea of paying for constrained news access (Kanuri et al., 2017).

These trends lead us to predict that the future of scarcity-driven monetization strategies for newspapers appears bright! The sustained economic viability of a monetization strategy for any firm revolves around a clear differentiation statement that resonates with its chosen customer segment(s). Local daily newspapers play an indispensable role in U.S. democracy by providing unique in-depth coverage of local policy issues (Ewens et al., 2022). In fact, local newspapers are especially well-positioned to carefully leverage their robust newsgathering infrastructure for delivering quality journalism, which can add sizable benefits to society that cannot be readily provided by other avenues (Turkel et al., 2021). Scarcity-based monetization strategies have a similar appeal. By leveraging the sound economic principles of second and third degree price discrimination, firms can now better differentiate “free” content from “premium” content–as a basis for “metering” readers' access to news as a function of their consumption. There is clear evidence that such a content differentiation strategy is appealing to ardent readers of news, even though this group may be small and niche. For instance, numerous academic studies report a higher propensity to pay for online content among readers that engage more with content. To the extent that readers continue subscribing to online news, and advertisers discover the upside of accessing an (arguably) higher willingness to pay and better engaged reader base, such price discrimination can supplement both subscription and advertising revenues. Furthermore, the adoption of scarcity-driven monetization strategies on their digital platforms can help newspapers improve their finances by lowering the cost of production and distribution of news.

On the other hand, non-scarcity based monetization strategies such as niche events and targeted fundraising appeals appear, so far, to offer limited appeal for making up for the stiff print subscription and advertising revenue losses experienced by newspapers. While the potential of such fundraising avenues seems promising for engaging and upselling current audiences, its ability to attract new subscribers is limited. Based on anecdotal reports, the economic viability of these events is a far cry in relation to the other revenue streams associated with the news product: news publishers worldwide rank advertising and subscription revenues as about twice or thrice as valuable as those from events and donations (eMarketer, 2019)^7^. Additionally, practitioners also question the revenue-generation potential of donation pleas targeted at readers who do not pay for news, raising further questions about the overall viability of non-scarcity based monetization strategies^8^. All of these trends seem to indicate that while newspapers may continue to adopt non-scarcity based news monetization strategies in the future to supplement their existing revenues, it is unlikely they can ever rely on them exclusively.

In fact, the Salt Lake Tribune even made an uncharacteristic switch to non-profit status to allow it to ward off hedge fund ownership that is becoming increasingly common in the market for news publishing. While some indicators suggest that such changes have allowed the Tribune to somewhat improve its immediate term financial stability, this is more an isolated example than a broader industry trend (Scire, 2021). Moreover, it is similarly unclear whether newspapers designed as non-profit will have better success with non-scarcity based fundraising appeals as a viable source of revenues.

In conclusion, while it may be challenging to expect that scarcity-based content monetization strategies will be viable enough to restore news publishers' finances to levels experienced during their glory days, recent trends in many newspapers' success with paywalls present an optimistic outlook for the sustainability of scarcity-based content monetization strategies for the industry. Producing quality content entails continued investment of both time and effort, and as a result, costs money. With the prevailing rapid spread of misinformation, society is increasingly likely to value rigorously vetted high quality journalism. At the same time, the flip side of scarcity-based content monetization strategies is it risks further dividing the society, based on differences in the ideological outlook of a small base of paying newspaper subscribers with access to trenchant news reporting, and the rest of society that risks exclusion from quality journalism on account of scarcity-based monetization. An optimal way forward for news publishers would involve balancing both their social welfare obligations and pecuniary objectives. There is little doubt that the world is a better place with quality journalism. Scarcity-based content monetization strategies seem both an inevitable and viable way forward for news publishers. With access to richer data on consumer demographics, political inclinations, beliefs and behaviors, publishers seem well-poised to incorporate further refinements in their paywall structures to alleviate the adverse consequences associated with the exclusion of “the masses” or of specific segments of society, from access to news. Our hope is that with scarcity-based monetization strategies, news organizations are not only able to remain profitable but also feel better inclined to fulfill their social obligations as stewards of a democratic society.

## Author contributions

All authors listed have made a substantial, direct, and intellectual contribution to the work and approved it for publication.

## Conflict of interest

The authors declare that the research was conducted in the absence of any commercial or financial relationships that could be construed as a potential conflict of interest.

## Publisher's note

All claims expressed in this article are solely those of the authors and do not necessarily represent those of their affiliated organizations, or those of the publisher, the editors and the reviewers. Any product that may be evaluated in this article, or claim that may be made by its manufacturer, is not guaranteed or endorsed by the publisher.

## Footnotes

^1^https://fitsmallbusiness.com/newspaper-advertising-costs/.

^2^Bisceglia, M. (2021). The unbundling of journalism. *SSRN*. 10.2139/ssrn.3885251

^3^https://www.aspenideas.org/.

^4^https://www.webmarketingpros.com/tablets-ebooks-on-the-rise-according-to-pew/-retrieved (accessed February 14, 2022).

^5^https://www.pewresearch.org/journalism/fact-sheet/newspapers/-retrieved (accessed February 14, 2022).

^6^Sen, A., and Yildirim, P. (2015). Clicks bias in editorial decisions: how does popularity shape online news coverage? *SSRN*. 10.2139/ssrn.2619440

^7^https://www.insiderintelligence.com/chart/225503/important-digital-revenue-streams-2019-according-news-publishers-worldwide-of-respondents (accessed February 14, 2022).

^8^For reference, less than 1% of Wikipedia's 450 million visitors donate money to the website–https://venturebeat.com/2015/07/27/with-less-than-1-of-its-users-giving-money-wikipedias-donation-ads-are-getting-bigger/-retrieved (accessed February 14, 2022)..
